# Stroke and suicide among people with severe mental illnesses

**DOI:** 10.1038/s41598-024-55564-x

**Published:** 2024-02-29

**Authors:** Chun-Hui Liao, Chen-Shu Chang, Pei-Tseng Kung, Wen-Yu Chou, Wen-Chen Tsai

**Affiliations:** 1 Department of Psychiatry, China Medical University Hospital, Taichung, Taiwan; 2 College of Medicine, China Medical University, Taichung, Taiwan; 3 Department of Neurology, Vascular and Genomic Research Center, Changhua Christian Hospital, Changhua, Taiwan; 4 Department of Medical Laboratory Science and Biotechnology, Central-Taiwan University of Science and Technology, Taichung, Taiwan; 5 Department of Healthcare Administration, Asia University, Taichung, Taiwan; 6 Department of Medical Research, China Medical University Hospital, China Medical University, Taichung, Taiwan; 7 Department of Health Services Administration, China Medical University, No. 100, Sec. 1, Jingmao Rd., Beitun Dist., Taichung, 406040 Taiwan

**Keywords:** Health care, Medical research

## Abstract

The associations between people with severe mental illnesses (SMI) and the risks of stroke, suicide, and death remain unclear. We examined healthcare service usage among adults with and without SMI and explored the risk of stroke, suicide, and death. We divided 18–80-year-old adults with SMI into catastrophic and non-catastrophic illness groups. These groups were subjected to a 1:5:5 propensity score matching with people without SMI. Data on demographic characteristics, economic factors, environmental factors, comorbid conditions, self-injury behavior, the number of outpatients and ED visits, and hospitalization were collected. The primary outcomes were risks of stroke, suicide, and death. We included 19,570 people with catastrophic SMI, 97,850 with non-catastrophic SMI, and 97,850 controls. Patients with SMI, especially those with catastrophic illnesses, had higher stroke risk, suicide, and death than those without SMI. People with SMI used health services more frequently than those without SMI. Patients with a history of hospitalization or ED access had a higher risk of stroke, suicide, and death. Our data indicate that special attention should be given to patients with SMI, particularly those with a history of healthcare service utilization, such as through more extended hospital stays with high-intensity interventions.

## Introduction

People with severe mental illness (SMI), including schizophrenia, bipolar disorder (BD), schizoaffective disorder, and major depressive disorder, have a disproportionately high mortality rate—two to three times that of the general population^1–3^. In Taiwan, these patients have a higher risk of death than the general population and significantly lower life expectancy at birth than the national standard (lower by 11.99–15.53 years in men and 6.83–15.48 years in women) ^4^, which may be due to natural or unnatural causes. Severe mental disorders are a well-established risk factor for suicidality^5^. More specifically, the lifetime risk of suicide is as high as 15% in people with affective disorders and 10% in those with schizophrenia. The risk of suicide in people with BD is 20–30 times higher than in the general population^6^.

Compared with suicide and accidental deaths, most people with SMI die from natural causes similar to those in the general population, including heart disease, cancer, cerebrovascular diseases, and lung and respiratory diseases, contributing the most to their years of lost life^7^. The findings revealed in the meta-analysis indicate that the elevated risk of premature death in individuals with BD cannot be solely attributed to suicide and unnatural causes; rather, somatic comorbidities also play a significant role ^8^. These SMI patients have poorer somatic health and often consume more healthcare resources. People with psychotic disorders had high levels of use of health services, both in absolute terms and relative to people without psychotic disorders^9^. According to a retrospective cohort study based on the population in Scotland, individuals with schizophrenia, BD, or depression exhibited a higher relative risk of stroke compared to those without psychiatric disorders^10^.

The National Health Insurance (NHI) program in Taiwan, implemented in 1995, provides most healthcare services to nearly 99.96% of the Taiwanese population^11^. Our objectives were to examine and explore the occurrence and associated factors of stroke, suicide, and mortality in individuals with SMI enrolled in the NHI program, as compared to a control group. Besides, patients who frequently visit the emergency department (ED) have a higher mortality rate than those who visit the ED less than once per year^12^. Moreover, compared with the general population, not only were people with ED visits more likely to die by suicide, but also were those with hospitalizations^13^. Hence, we additionally aimed to comprehend the utilization of health services among individuals in the Taiwanese population who experienced stroke, suicide, and mortality.

## Results

After 1:5:5 matching, 215,270 patients were included: 19,570 in the catastrophic SMI group, 97,850 in the SMI group, and 97,850 in the control group. Their characteristics are presented in Table 1. The mean age of the three groups was approximately 41 years. The distributions of sex, age groups, and CCI were not statistically different among the three groups after matching. In addition, the SMI groups had lower education levels, a lower proportion of married individuals, and lower incomes. Moreover, compared with the control group, the SMI groups had a higher percentage of patients with self-harm behavior, use of outpatient department (OPD) medical services, emergency services, and hospital admissions.

**Table 1:** Baseline characteristics of people with severe mental illness (SMI) with catastrophic illness certificate, matched people with SMI but no catastrophic illness certificate and matched people without SMI.

Variables	SMI with catastrophic illness certificate		SMI without catastrophic illness certificate		No SMI group		*p*-value^&^
	n1	%	n2	%	n3	%	
Total	19,570	9.09	97,850	45.45	97,850	45.45	
Sex							0.813
Male	8439	43.12	42,291	43.22	42,195	43.12	
Female	11,131	56.88	55,559	56.78	55,655	56.88	
Age							1.000
≦24	2194	11.21	10,965	11.21	10,970	11.21	
25–34	4902	25.05	24,476	25.01	24,510	25.05	
35–44	4852	24.79	24,220	24.75	24,260	24.79	
45–54	4246	21.70	21,265	21.73	21,230	21.70	
55–64	2457	12.55	12,329	12.60	12,285	12.55	
≧65	919	4.70	4595	4.70	4595	4.70	
Mean ± SD	40.96 ± 13.29		41.14 ± 13.58		41.07 ± 13.53		0.190
CCI score							0.979
0	15,381	78.59	76,895	78.58	76,905	78.59	
1	2968	15.17	14,910	15.24	14,840	15.17	
2	856	4.37	4277	4.37	4280	4.37	
≧3	365	1.87	1768	1.81	1825	1.87	
Education level							< 0.001
Elementary or below	3441	17.58	15,721	16.07	14,934	15.26	
Junior high	5778	29.52	24,681	25.22	21,134	21.60	
Senior high	7992	40.84	40,574	41.47	39,497	40.36	
College or above	2359	12.05	16,874	17.24	22,285	22.77	
Marital status							< 0.001
Unmarried	8603	43.96	31,521	32.21	31,659	32.35	
Married	6884	35.18	50,764	51.88	56,592	57.84	
Divorce	3432	17.54	12,584	12.86	6534	6.68	
Death of spouse	651	3.33	2981	3.05	3065	3.13	
Monthly salary (NTD)							< 0.001
≦21,009	8138	41.58	36,723	37.53	34,884	35.65	
21,010–22,800	541	2.76	3012	3.08	3167	3.24	
22,801–28,800	7610	38.89	27,343	27.94	22,696	23.19	
28,801–36,300	1206	6.16	9684	9.90	11,312	11.56	
36,301–45,800	1118	5.71	9556	9.77	11,598	11.85	
45,801–57,800	443	2.26	4670	4.77	5956	6.09	
≧57,801	514	2.63	6862	7.01	8237	8.42	
Urbanization level							< 0.001
Level 1	5438	27.79	28,922	29.56	29,958	30.62	
Level 2	6654	34.00	32,594	33.31	31,365	32.05	
Level 3	3027	15.47	16,360	16.72	17,267	17.65	
Level 4	2403	12.28	12,303	12.57	11,899	12.16	
Level 5	315	1.61	1439	1.47	1416	1.45	
Level 6	1040	5.31	3130	3.20	2904	2.97	
Level 7	693	3.54	3102	3.17	3041	3.11	
Self-harm behavior in the previous year before the index date							< 0.001
No	19,443	99.35	97,644	99.79	97,849	100.00	
Yes	127	0.65	206	0.21	1	0.00	
Outpatient visits in the previous year before the index date							< 0.001
≦Q1	3607	18.43	15,749	16.10	38,624	39.47	
Q1–Q2	3441	17.58	21,959	22.44	26,051	26.62	
Q2–Q3	5168	26.41	27,309	27.91	20,770	21.23	
> Q3	7354	37.58	32,833	33.55	12,405	12.68	
Mean ± SD	26.21 ± 23.74		24.45 ± 20.80		13.22 ± 13.75		
Emergency department visits in the previous year before the index date							< 0.001
0	10,311	52.69	65,572	67.01	84,337	86.19	
1 time	4508	23.04	18,763	19.18	10,417	10.65	
≧2 times	4751	24.28	13,515	13.81	3096	3.16	
Mean ± SD	1.22 ± 2.96		0.66 ± 1.78		0.19 ± 0.60		
Hospitalization in the previous year before the index date							< 0.001
No	9207	47.05	81,279	83.06	92,069	94.09	
Yes	10,363	52.95	16,571	16.94	5781	5.91	
Suicide							< 0.001
No	19,080	97.50	96,696	98.82	97,761	99.91	
Yes	490	2.50	1154	1.18	89	0.09	

^&^χ^2^ test; Index date: the date with newly diagnosed depression, bipolar disorder, or schizophrenia for the first time from 2008 to 2010.

Table 2 presents the differences among the three groups by bivariate analysis of the log-rank test. Table 3 shows the results of the Cox proportional hazards model. After adjustment for the other variables, the highest risk of stroke was noted in the SMI group (HR = 1.33, 95% CI 1.22–1.45, *p* < 0.001), followed by the catastrophic SMI group (HR = 1.20, 95% CI 1.06–1.36, *p* = 0.004). The catastrophic SMI group had the highest risk of suicide (HR = 20.53, 95% CI 15.50–27.19, *p* < 0.001) and death (HR = 2.60, 95% CI 2.41–2.80, *p* < 0.001; Table 3) comparing with those without SMI.

**Table 2:** The distribution of stroke, suicide, and death among patients with severe mental illness (SMI) with catastrophic illness certificate, matched people with SMI but no catastrophic illness certificate, and matched people without SMI.

Variables	Stroke				*p*^&^	Suicide				*p*^&^	Death				*p*^&^
	No		Yes			No		Yes			No		Yes		
	n	%	n	%		n	%	n	%		n	%	n	%	
Total	210,896	97.97	4374	2.03		213,537	99.19	1733	0.81		204,970	95.22	10,300	4.78	
Severity of mental illness					< 0.001					< 0.001					< 0.001
No SMI group	96,237	98.35	1613	1.65		97,761	99.91	89	0.09		94,946	97.03	2904	2.97	
SMI without catastrophic illness certificate	95,586	97.69	2264	2.31		96,696	98.82	1154	1.18		92,372	94.40	5478	5.60	
SMI with catastrophic illness certificate	19,073	97.46	497	2.54		19,080	97.50	490	2.50		17,652	90.20	1918	9.80	
Sex					< 0.001					< 0.001					
Male	90,711	97.62	2214	2.38		91,960	98.96	965	1.04		86,789	93.40	6136	6.60	
Female	120,185	98.23	2160	1.77		121,577	99.37	768	0.63		118,181	96.60	4164	3.40	
Age					< 0.001					< 0.001					< 0.001
≦24	24,081	99.80	48	0.20		23,970	99.34	159	0.66		23,831	98.76	298	1.24	
25–34	53,669	99.59	219	0.41		53,402	99.10	486	0.90		52,555	97.53	1333	2.47	
35–44	52,756	98.92	576	1.08		52,860	99.11	472	0.89		51,421	96.42	1911	3.58	
45–54	45,630	97.62	1111	2.38		46,381	99.23	360	0.77		44,506	95.22	2235	4.78	
55–64	25,744	95.10	1327	4.90		26,895	99.35	176	0.65		25,013	92.40	2058	7.60	
≧65	9016	89.19	1093	10.81		10,029	99.21	80	0.79		7644	75.62	2465	24.38	
Mean ± SD	40.78 ± 13.37		55.90 ± 13.00		< 0.001	41.09 ± 13.54		40.61 ± 12.76		0.137	40.54 ± 13.17		52.10 ± 15.74		< 0.001
CCI Score					< 0.001					0.011					< 0.001
0	166,776	98.58	2405	1.42		167,806	99.19	1375	0.81		163,178	96.45	6003	3.55	
1	31,648	96.73	1070	3.27		32,477	99.26	241	0.74		30,470	93.13	2248	6.87	
2	8814	93.64	599	6.36		9342	99.25	71	0.75		8256	87.71	1157	12.29	
≧3	3658	92.42	300	7.58		3912	98.84	46	1.16		3066	77.46	892	22.54	
Education level					< 0.001					< 0.001					< 0.001
Elementary or below	32,135	94.25	1961	5.75		33,804	99.14	292	0.86		30,329	88.95	3767	11.05	
Junior high	50,665	98.20	928	1.80		51,030	98.91	563	1.09		48,880	94.74	2713	5.26	
Senior high	86,934	98.72	1129	1.28		87,381	99.23	682	0.77		85,057	96.59	3006	3.41	
College or above	41,162	99.14	356	0.86		41,322	99.53	196	0.47		40,704	98.04	814	1.96	
Marital status					< 0.001					< 0.001					< 0.001
Unmarried	71,285	99.31	498	0.69		71,141	99.11	642	0.89		69,387	96.66	2396	3.34	
Married	111,374	97.49	2866	2.51		113,565	99.41	675	0.59		108,919	95.34	5321	4.66	
Divorce	21,998	97.55	552	2.45		22,170	98.31	380	1.69		20,894	92.66	1656	7.34	
Death of spouse	6239	93.16	458	6.84		6661	99.46	36	0.54		5770	86.16	927	13.84	
Monthly salary (NTD)					< 0.001					< 0.001					< 0.001
≦21,009 NTD	77,874	97.65	1871	2.35		79,094	99.18	651	0.82		75,401	94.55	4344	5.45	
21,010–22,800	6624	98.57	96	1.43		6671	99.27	49	0.73		6495	96.65	225	3.35	
22,801–28,800	56,330	97.71	1319	2.29		56,981	98.84	668	1.16		53,986	93.65	3663	6.35	
28,801–36,300	21,852	98.42	350	1.58		22,080	99.45	122	0.55		21,501	96.84	701	3.16	
36,301–45,800	21,896	98.31	376	1.69		22,155	99.47	117	0.53		21,610	97.03	662	2.97	
45,801–57,800	10,939	98.83	130	1.17		11,012	99.49	57	0.51		10,770	97.30	299	2.70	
≧57,801	15,381	98.51	232	1.49		15,544	99.56	69	0.44		15,207	97.40	406	2.60	
Urbanization level					< 0.001					0.131					< 0.001
Level 1	63,247	98.33	1071	1.67		63,838	99.25	480	0.75		61,884	96.22	2434	3.78	
Level 2	69,241	98.06	1372	1.94		70,045	99.20	568	0.80		67,465	95.54	3148	4.46	
Level 3	35,935	98.04	719	1.96		36,355	99.18	299	0.82		34,913	95.25	1741	4.75	
Level 4	25,922	97.43	683	2.57		26,384	99.17	221	0.83		25,054	94.17	1551	5.83	
Level 5	3072	96.91	98	3.09		3140	99.05	30	0.95		2922	92.18	248	7.82	
Level 6	6823	96.45	251	3.55		7007	99.05	67	0.95		6402	90.50	672	9.50	
Level 7	6656	97.37	180	2.63		6768	99.01	68	0.99		6330	92.60	506	7.40	
Self-harm behavior one year before the index date					0.239					< 0.001					< 0.001
No	210,571	97.97	4365	2.03		213,228	99.21	1708	0.79		204,680	95.23	10,256	4.77	
Yes	325	97.31	9	2.69		309	92.51	25	7.49		290	86.83	44	13.17	
Outpatient visits in the previous year before the index date					< 0.001					< 0.001					< 0.001
≦Q1	57,260	98.76	720	1.24		57,589	99.33	391	0.67		55,813	96.26	2167	3.74	
Q1–Q2	50,762	98.66	689	1.34		51,087	99.29	364	0.71		49,618	96.44	1833	3.56	
Q2–Q3	52,160	97.96	1087	2.04		52,837	99.23	410	0.77		50,841	95.48	2406	4.52	
> Q3	50,714	96.43	1878	3.57		52,024	98.92	568	1.08		48,698	92.60	3894	7.40	
Mean ± SD	19.31 ± 18.96		29.21 ± 25.22			19.46 ± 19.09		25.20 ± 25.64			19.13 ± 18.65		27.04 ± 26.22		
Emergency department visits in the previous year before the index date					< 0.001					< 0.001					< 0.001
No	157,408	98.24	2812	1.76		159,355	99.46	865	0.54		154,138	96.20	6082	3.80	
1 time	32,863	97.55	825	2.45		33,274	98.77	414	1.23		31,626	93.88	2062	6.12	
≧2 times	20,625	96.55	737	3.45		20,908	97.87	454	2.13		19,206	89.91	2156	10.09	
Mean ± SD	0.49 ± 1.57		0.84 ± 2.17			0.49 ± 1.57		1.29 ± 2.44			0.46 ± 1.46		1.13 ± 3.07		
Hospitalization in the previous year before the index date			< 0.001												< 0.001
No	179,351	98.24	3204	1.76		181,467	99.40	1088	0.60		175,795	96.30	6760	3.70	
Yes	31,545	96.42	1170	3.58		32,070	98.03	645	1.97		29,175	89.18	3540	10.82	
Suicide					0.013					< 0.001					< 0.001
No	209,186	97.96	4351	2.04		–	–	–	–		204,970	95.99	8567	4.01	
Yes	1710	98.67	23	1.33		–	–	–	–		0	0.00	1733	100.00	

^&^Log-rank test.

**Table 3:** Risk factors of stroke, suicide, and death among patients with severe mental illness (SMI) with catastrophic illness certificate, matched people with SMI but no catastrophic illness certificate, and matched people without SMI by Cox Proportional Hazard Model.

Variables	Stroke				Suicide				Death			
	Adjusted HR	95% C.I		*p*-value^&^	Adjusted HR	95% C.I		*p*-value^&^	Adjusted HR	95% C.I		*p*-value^&^
Research groups												
No SMI group (ref.)	1.00				1.00				1.00			
SMI without catastrophic illness certificate	1.33	1.22	1.45	< 0.001	12.87	9.91	16.71	< 0.001	2.04	1.92	2.16	< 0.001
SMI with catastrophic illness certificate	1.20	1.06	1.36	0.004	20.53	15.50	27.19	< 0.001	2.60	2.41	2.80	< 0.001
Sex												
Male (ref.)	1.00				1.00				1.00			
Female	0.53	0.48	0.60	< 0.001	0.38	0.26	0.55	< 0.001	0.40	0.37	0.43	< 0.001
Age												
≦ 24 (ref.)	1.00				1.00				1.00			
25–34	1.98	1.33	2.97	< 0.001	2.00	1.06	3.77	0.033	2.78	2.25	3.44	< 0.001
35–44	6.15	4.19	9.03	< 0.001	1.96	1.05	3.66	0.034	4.94	4.03	6.06	< 0.001
45–54	11.99	8.10	17.74	< 0.001	1.95	1.01	3.74	0.046	8.00	6.46	9.92	< 0.001
55–64	21.82	14.56	32.71	< 0.001	2.07	0.96	4.45	0.064	14.08	11.19	17.71	< 0.001
≧ 65	40.39	25.54	63.87	< 0.001	5.25	1.36	20.34	0.016	35.60	27.02	46.89	< 0.001
CCI score												
0 (ref.)	1.00				1.00				1.00			
1	1.27	1.12	1.44	< 0.001	0.84	0.55	1.29	0.418	1.28	1.18	1.40	< 0.001
2	1.79	1.45	2.21	< 0.001	0.69	0.33	1.41	0.306	1.66	1.45	1.91	< 0.001
≧3	2.24	1.64	3.06	< 0.001	3.67	1.46	9.26	0.006	2.97	2.47	3.57	< 0.001
Education level												
Elementary or below (ref.)	1.00				1.00				1.00			
Junior high	0.83	0.75	0.91	< 0.001	1.04	0.86	1.25	0.720	0.97	0.91	1.04	0.409
Senior high	0.66	0.60	0.72	< 0.001	0.77	0.64	0.93	0.006	0.69	0.65	0.74	< 0.001
College or above	0.47	0.41	0.54	< 0.001	0.60	0.48	0.76	< 0.001	0.45	0.41	0.50	< 0.001
Marital status												
Unmarried (ref.)	1.00				1.00				1.00			
Married	0.98	0.87	1.10	0.723	0.81	0.69	0.94	0.007	0.68	0.63	0.72	< 0.001
Divorce	1.25	1.08	1.44	0.002	1.47	1.24	1.74	< 0.001	1.13	1.04	1.22	0.003
Death of spouse	1.26	1.07	1.49	0.005	0.65	0.44	0.98	0.041	0.98	0.88	1.08	0.632
Monthly salary (NTD)												
≦21,009 (ref.)	1.00				1.00				1.00			
21,010–22,800	0.75	0.60	0.94	0.011	0.99	0.72	1.37	0.958	0.86	0.74	0.99	0.035
22,801–28,800	1.01	0.94	1.10	0.736	1.26	1.11	1.43	< 0.001	1.17	1.11	1.23	< 0.001
28,801–36,300	0.75	0.66	0.85	< 0.001	0.84	0.68	1.04	0.118	0.80	0.73	0.87	< 0.001
36,301–45,800	0.75	0.66	0.85	< 0.001	0.91	0.73	1.14	0.415	0.72	0.65	0.78	< 0.001
45,801–57,800	0.70	0.57	0.84	< 0.001	0.97	0.71	1.31	0.827	0.81	0.72	0.92	0.001
≧57,801	0.78	0.67	0.90	0.001	0.85	0.65	1.13	0.262	0.73	0.65	0.81	< 0.001
Urbanization level												
Level 1 (ref.)	1.00				1.00				1.00			
Level 2	1.07	0.98	1.16	0.145	1.04	0.91	1.20	0.554	1.09	1.03	1.15	0.005
Level 3	1.09	0.98	1.20	0.115	1.07	0.91	1.26	0.398	1.15	1.07	1.23	< 0.001
Level 4	1.15	1.03	1.28	0.014	0.98	0.81	1.17	0.789	1.17	1.09	1.26	< 0.001
Level 5	1.10	0.87	1.39	0.419	1.12	0.73	1.72	0.591	1.30	1.12	1.51	< 0.001
Level 6	1.08	0.93	1.27	0.316	1.04	0.77	1.40	0.799	1.30	1.18	1.44	< 0.001
Level 7	0.97	0.81	1.16	0.736	1.21	0.90	1.63	0.206	1.26	1.13	1.41	< 0.001
Self-harm behavior one year before the index date												
No (ref.)					1.00							
Yes	–	–	–	–	3.12	1.72	5.67	< 0.001	–	–	–	–
Outpatient visits in the previous year before the index date												
≦Q1 (ref.)	1.00				1.00				1.00			
Q1–Q2	0.95	0.85	1.07	0.401	0.87	0.74	1.03	0.104	0.83	0.78	0.89	< 0.001
Q2–Q3	1.00	0.90	1.12	0.991	0.85	0.72	1.00	0.051	0.78	0.73	0.83	< 0.001
> Q3	0.99	0.88	1.10	0.809	0.90	0.76	1.07	0.225	0.72	0.67	0.77	< 0.001
Emergency department visits in the previous year before the index date												
No (ref.)	1.00				1.00				1.00			
1 time	1.24	1.13	1.35	< 0.001	1.44	1.25	1.65	< 0.001	1.23	1.16	1.30	< 0.001
≧2 times	1.44	1.30	1.59	< 0.001	1.74	1.50	2.01	< 0.001	1.61	1.51	1.71	< 0.001
Hospitalization in the previous year before the index date												
No (ref.)	1.00				1.00				1.00			
Yes	1.45	1.33	1.58	< 0.001	1.44	1.26	1.65	< 0.001	1.62	1.54	1.71	< 0.001

^&^Conditional Cox proportional hazard model.

According to the variables in the adjusted model (Table 3), women were associated with a lower risk of stroke (HR = 0.53, 95% CI 0.48–0.60, *p* < 0.001), suicide (HR = 0.38, 95% CI 0.26–0.55, *p* < 0.001), and death (HR = 0.40, 95% CI 0.37–0.43, *p* < 0.001). Moreover, a trend of increasing stroke and death risk, but not suicide risk, was observed with an increase in age. Older adults (≥ 65 years) had a 40.39-fold risk of stroke and a 5.25-fold risk of suicide compared with young adults (≤ 24 years).

Compared with people without comorbidities, the higher the CCI, the higher the stroke risk (CCI ≥ 3: HR = 2.24, followed by CCI = 2: HR = 1.79, and CCI = 1: HR = 1.27). After controlling for all other variables, individuals with education levels higher than senior high school had a 0.45–0.6-fold (95% CI 0.41–0.76) lower risk of stroke, suicide, and death. Divorced people had the highest risk of suicide (HR = 1.47, 95% CI 1.24–1.74, *p* < 0.001) and death (HR = 1.13, 95% CI 1.04–1.22, *p* = 0.003) compared with other individuals. People whose monthly salary was NT$22,808–28,800 had the highest risk of suicide and death (HR = 1.26, 95% CI 1.11–1.43; HR = 1.17, 95% CI 1.11–1.23) (Table 3).

A history of self-harm was associated with a higher risk of suicide (HR = 3.12, 95% CI 1.72–5.67, *p* < 0.001). A higher frequency of outpatient visits was associated with a lower risk of death. ED visits and admission also were associated with the risk of stroke, suicide, and death (Table 3). According to the univariate Poisson regression test, the catastrophic SMI group had the highest incidence of stroke, suicide, and death (3.13, 3.05, and 11.94 per 1000 person-years, respectively), followed by the SMI group (2.79, 1.41, and 6.68 per 1000 person-years, respectively; Table 4).

**Table 4:** Incidence per thousand person-years of stroke, suicide, and death among patients with severe mental illness (SMI) with catastrophic illness certificate, matched patients with SMI but no catastrophic illness certificate, and matched people without SMI.

Research groups	Stroke	Total person-years	Incidence rate/1000 person-years	*p*-value^&^
No SMI group	1613	875,853	1.84	–
SMI without catastrophic illness certificate	2264	811,516	2.79	< 0.001
SMI with catastrophic illness certificate	497	158,705	3.13	< 0.001

Research groups	Suicide	Total person-years	Incidence rate/1000 person-years	*p*-value^&^
No SMI group	89	881,532	0.10	–
SMI without catastrophic illness certificate	1154	819,629	1.41	< 0.001
SMI with catastrophic illness certificate	490	160,579	3.05	< 0.001

Research groups	Death	Total person-years	Incidence rate/1000 person-years	*p*-value^&^
No SMI group	2904	881,532	3.29	–
SMI without catastrophic illness certificate	5478	819,629	6.68	< 0.001
SMI with catastrophic illness certificate	1918	160,579	11.94	< 0.001

^&^Univariate Poisson regression test for Poisson distribution.

Supplementary Figures A, B, and C present the covariate-adjusted cumulative incidence curves of stroke, suicide, and death among people with SMI compared with the control group according to the Cox proportional hazard model.

## Discussion

In the current nationwide cohort study, we observed higher rates of stroke, suicide, and death in patients with SMI than in those without SMI. Patients with SMI with catastrophic illness certificates were at the highest risk. Additionally, people with SMI used health services more frequently than those without SMI. Patients hospitalized or accessed ED services were at higher risk of stroke, suicide, and death. However, patients with higher outpatient use had a lower risk of death, with no effect on stroke and suicide. Our results are similar to another population-based cohort study from New Zealand, which reported an association between psychiatric disorders and self-harm behavior^14^. Mental disorders were associated with the onset of physical illness, multiple physical illness diagnoses, higher hospitalizations, and early mortality^14^.

Our data indicated that SMI was associated with a higher risk of stroke, consistent with several studies^15,16^. A meta-analysis of six cohort studies confirmed a modest but significant positive association between schizophrenia and stroke morbidity and mortality^17^; patients with BD had a significantly increased risk of stroke^18^. Numerous mechanisms have been proposed to explain the association between SMI and stroke. For instance, SMI correlates with adverse effects of drug therapy^19^ and behavioral alterations such as smoking^20^, inadequate physical activity, and insufficient dietary/caloric intake^21^. These behavioral changes, in turn, are linked to the etiology of diverse diseases, including diabetes and cardiovascular disease, which could serve as contributors to the occurrence of stroke and premature mortality. In addition, insufficient health care availability^22^ may increase natural-cause mortality in psychiatric patients. Disparities in mortality rates and reduced life expectancy serve as markers of health inequalities, and people with mental illness do not benefit equally from social and healthcare advancements experienced by the general population^23^. Because of the NHI program in Taiwan, in our study, patients with SMI had higher rates of medical service use than the controls, indicating no barriers to using medical services. Nonetheless, our data lacks specific information on medical care details and quality. This is our limitation in displaying the purpose of the medical service visit and the service quality. Besides, people with SMI are less likely to receive preventive care services (such as screening for cardiovascular risk factors) and high-quality care than those without SMI^24^. Moreover, certain research findings suggest challenges for individuals with SMI in effectively managing their chronic conditions^25^. Schizophrenia is correlated with a diminished likelihood of receiving high-quality diabetes care, contributing to an elevated risk of diabetes-related emergency department visits and hospitalizations^26^. Notably, there is a notable deficiency in the prescription of various common medications, particularly those addressing cardiovascular issues, in individuals with SMI, including those diagnosed with schizophrenia^27^. Thus, considerable gaps remain in routine health care for many people with mental illness^24^. These gaps include physicians focusing on mental illness rather than physical health, inconsistent adherence to health checks and treatment, and poor communication. Patients with SMI tend to have lower socioeconomic backgrounds, which may make it more difficult for them to use medical resources appropriately^23^. Taiwan’s psychiatric medical care is highly developed and covered under the NHI, enabling people to seek treatment in community psychiatric clinics or hospitals; they can also go to other departments to receive medical treatment for physical diseases. In our study, we found that a relatively high proportion of older patients were first diagnosed with SMI between 2008 and 2011, considering the age at which SMI is most likely to occur. Our research relies on observations sourced from the National Health Insurance database, and the observed phenomenon might be attributed to the substantial time lapse between the onset and formal diagnosis of SMI. Diagnostic delays can stem from various factors, including patients' limited insight or societal taboos discouraging them from seeking medical treatment. Additionally, some instances arise from healthcare professionals downplaying the diagnosis of SMI and instead categorizing milder mental conditions as the primary diagnosis^28^.

We observed no increased suicide risk in new-onset strokes in patients with SMI. Selective survival and competing causes of death may explain this pattern. Individuals with lower socioeconomic status may die earlier for other reasons so that only the healthiest survive into old age^29^. It has been suggested that higher rates of selective survival among vulnerable, high-mortality populations lead to higher proportions of healthy older individuals who may have lower suicide rates. Because stroke is more common in old age^30^, those with SMI surviving into old age who develop stroke may not be at increased suicide risk.

In addition to a high risk of death from natural causes, SMI has also been associated with a higher risk of suicide^6,31^, consistent with our results. Furthermore, some studies have concluded that older adults have the highest suicide rates^32^, which also agrees with our data. In our analysis, a CCI score of ≥ 3 was associated with the highest suicide risk, in line with other studies^33^. Furthermore, consistent with Gallego et al. ^34^, we observed that a history of suicide attempts was associated with higher suicide rates. This finding has been corroborated by large systematic reviews by Beghi et al.^35^, 76 studies, and Larkin et al.^36^, 129 studies. Therefore, patients aged ≥ 65 with SMIs, new arrivals to local mental health services, patients with multiple chronic diseases, divorced or widowed individuals, and those with low education levels should receive multidimensional assistance.

Studies have attempted to predict suicidal behavior from electronic health records. The factors predicting suicide include post-psychiatric hospitalization^37^, prediction of suicide or accidental death following civilian general hospital discharge^38^, and prediction of suicide attempt or suicide following outpatient visits. For example, Barak-Corren and colleagues^39^ used health records data from two large academic health systems to predict suicide attempts or deaths among outpatients with ≥ 3 visits. In our analysis, we observed that suicide cases had a higher rate of ED visits, hospitalization, and previous self-harm records. These characteristics may be helpful for the prediction of suicide in the Taiwanese medical service system.

Given this clinically significant overlap, patients with mental illness should be asked if they have used medical services adequately. It remains unclear what proportion of physical health needs and treatment is unmet in this population compared with individuals without documented mental illness. However, epidemiologic research in community and clinical settings reveals a strong association between mental disorders and increased healthcare service utilization^40^. Psychiatric disorders are often associated with frequent outpatient services in Taiwan^41^. Analytical results do not replace clinical judgment, but risk scores can inform individual clinical decision-making and quality improvement plans. Our results may help provide a risk score to help determine risk stratification. We will also suggest further studies related to “mortality risk factors associated with severe mental illnesses” and “reasons associated with emergency department visits and hospitalizations for SMI patients.”

### Strengths and limitations

This study used a national population database to avoid selection and participation bias^42^. Other strengths include the large sample size and number of events and the long follow-up time, which increase the likelihood of finding significance even if the differences are slight; therefore, all results should be interpreted in terms of the clinical importance of the differences and provide strong evidence for understanding possible factors in the real world.

This study has several limitations. First, the NHIRD lacks data on important confounders for increased stroke and mortality, including smoking, dietary habits, and physical activity, precluding the adjustment for these confounders in our regression models. Second, health system records do not reflect crucial social risk factors for suicidal behavior, such as unemployment, bereavement, or relationship breakdown. Suicidal behavior may reflect an interaction of clinical risk factors, adverse life events, and available means of self-harm. Moreover, the monthly income is based on NHI premium–based salary and not actual income data. Third, no information is available on the severity of illness at the time of suicide. The lack of seriousness and clinical status of SMI precludes the determination of the association between mental status and outcome. However, we tried to use the catastrophic disease system to help define severity. Our results are still valid as a reference for clinical care. Fourth, we lack information on substance abuse. The prevalence of comorbidity between SMI and substance use disorder (SUD) is substantial, with estimates indicating that up to 75% of SMI patients also have SUD, and approximately 60% of adults with SUD exhibit some form of SMI. The presence of coexisting disorders is associated with an increased likelihood of adverse health outcomes, suicide, unplanned hospital admissions, and premature mortality^43^.

### Clinical implications

The higher rates of stroke, suicide, and death in our cohort of patients with SMI suggest that special attention should be given to their health and well-being and strategies to improve them. Our data indicate that the higher the ED utilization and hospitalization rate, the higher the risk of suicide, stroke, and death. These findings highlight opportunities to improve both disease and suicide prevention. A significant effort to prevent suicide in all patients with SMI in the ED appears warranted, especially among those with self-harming behaviors, multiple chronic diseases, and high ED utilization. Hospitalization is also a risk of death by suicide compared with people who are not hospitalized. The period of hospitalization provides an opportunity for potentially high-intensity interventions. When dealing with patients with SMI, clinicians should consider more extended hospital stays, which may help initiate high-intensity physical and mental health interventions, before discharging them to outpatient care. Additionally, it is crucial for clinicians to prioritize individuals with high ED and hospitalization utilization, as they may harbor potential SMI cases. Developing a model for case management could aid in enhancing the health condition of these patients and potentially reduce the overutilization of medical services.

## Materials and methods

### Data sources

This nationwide population-based cohort study used data from Taiwan’s National Health Insurance Research Database (NHIRD)^44^. In addition, in this study, we used the Longitudinal Health Insurance Database, the Taiwan Cause of Death Statistics, and the Household Registration File managed by the Ministry of the Interior. These databases were provided by the Health and Welfare Data Science Center under the Ministry of Health and Welfare (MOHW). Personally identifiable information was de-identified before its release. Therefore, the collected data comply with personal data protection regulations.

### Study population

The data of patients aged 18–80 who received the first diagnosis of SMI between 2008 and 2010 were extracted from the NHIRD. SMI was identified through International Classification of Diseases, Ninth Revision, Clinical Modification (ICD-9-CM) codes as follows: at least one instance of hospitalization or ≥ three outpatient visits within 365 days with the diagnosis of depression (ICD-9-CM: 296.2X, 296.3X, 300.4X), BD (ICD-9-CM: 296.XX, excluding 296.2X and 296.3X), or schizophrenia (ICD-9-CM: 295.XX), respectively.

This study divided patients with SMI into catastrophic and non-catastrophic illness groups. The index date for these two groups was the date of the new diagnosis, and that for the control group was the first day of the matching year. The catastrophic illness group comprised patients a physician diagnosed as having a “catastrophic illness” and a catastrophic illness certificate dated between 2008 and 2010. People with serious illnesses who met the National Health Insurance’s definition of catastrophic illnesses as diagnosed by physicians, including 30 categories of patients such as cancers, chronic mental illness, chronic renal failure, type I diabetes, autoimmune disease, congenital factor disorder, stroke, congenital hypothyroidism, etc. Patients with catastrophic illnesses were exempted from copayment and thus avoided financial burden for its long-term health care. Psychiatric conditions such as schizophrenia, schizoaffective disorder, BD, and major depressive disorder may be considered. In cases where a patient exhibits a decline in occupational function, the physician assists them in obtaining a certificate for catastrophic illnesses. Patients with a catastrophic illness certificate receive care for a disease or related condition within the certificate’s validity period, without paying out-of-pocket costs for outpatient or inpatient treatment. However, these patients must follow standard treatment and payment procedures when seeking care for unrelated illnesses^45^.

**Figure 1:**
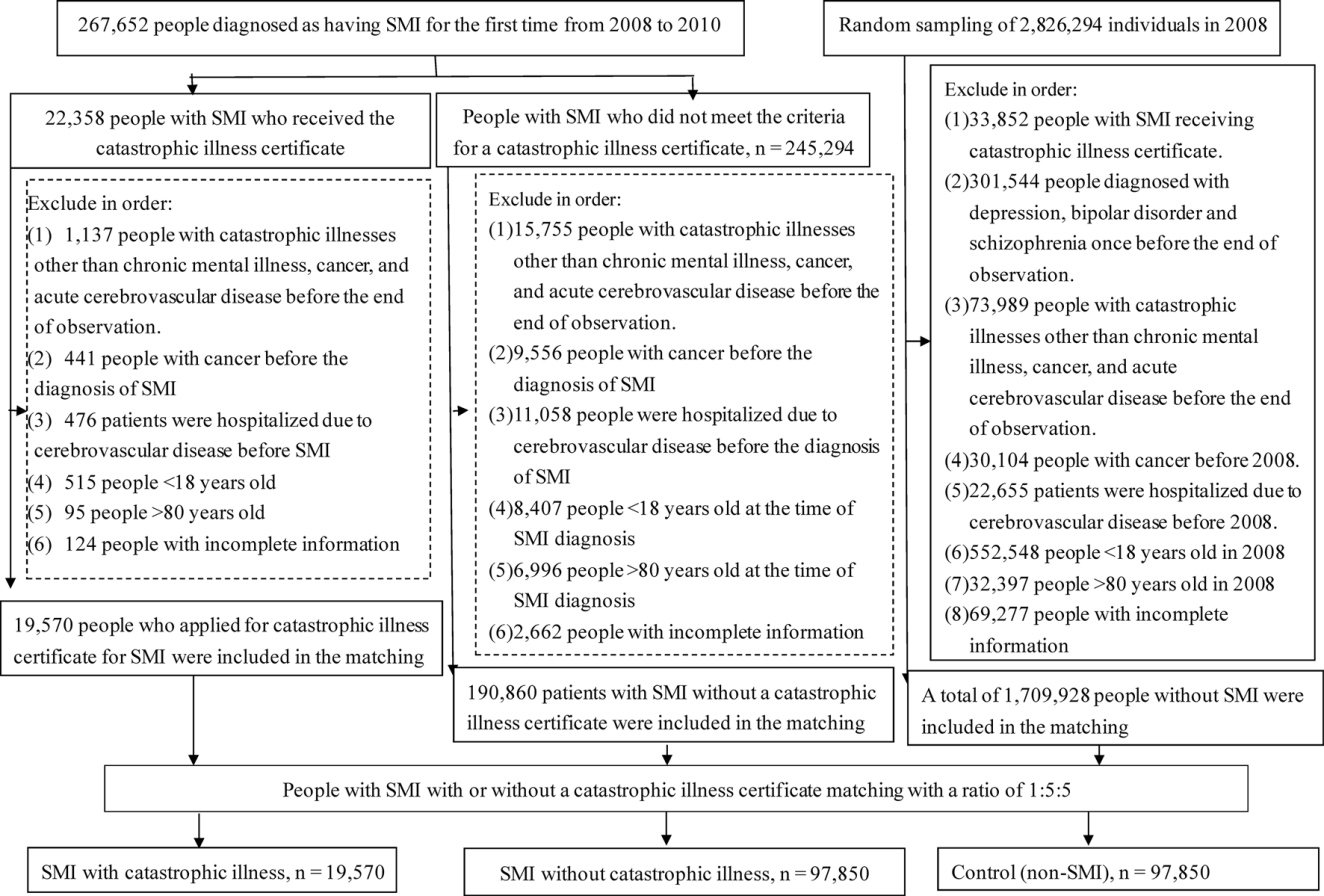
Flowchart of patient selection. SMI: severe mental illness (depression, bipolar disorder, or schizophrenia).

For the control group, 3 million cases were randomly selected from the National Health Insurance Beneficiaries File, and those with a primary or secondary diagnosis of SMI from 2000 to 2017 were excluded. To reduce substantial differences in patient characteristics between the three groups, a 1:5:5 propensity score matching was conducted among the catastrophic illness, illness, and control groups year by year according to sex, age, and severity of comorbidities, thereby decreasing the selection bias. A greedy nearest neighbor algorithm was used for matching. In addition, we excluded individuals with a diagnosis of any catastrophic illness other than depression, BD, and schizophrenia, as well as those diagnosed as having cancer or stroke before the index year. The patient selection flowchart is presented in Fig. 1.

### Definition of variables

In this study, the following variables were included: (a) Demographic characteristics: sex, age, education level, and marital status. (b) Economic factors: monthly salary divided into seven bands. (c) Environmental factors: urbanization level of the patients’ areas of residence, with the 359 townships, cities, and districts across Taiwan categorized from level 1 (most urban) to level 7 (most rural)^46^. (d) Comorbid conditions: The comorbidity severity was used as an indicator of health status and was measured using the modified version of the Charlson comorbidity index (CCI) by Deyo et al.^47^. based on the CCI scores calculated using medical records up to 2 years before the index date, the sample was divided into four groups (0, 1, 2, and ≥ 3). (e) Healthcare service access: We observed some of the medical situations of the research participants in the year before the index date, including self-harm behavior, the number of outpatient visits, the number of ED visits, and the history of hospitalization. The number of outpatient visits was divided into quartiles: ≤ Q1, Q1–Q2, Q2–Q3, and > Q3.

### Main outcomes and comorbidities

We analyzed the risk of stroke, suicide, and death in people with SMI from the index date through the end of 2017. Self-harm behavior (ICD-9 codes E950–E959 and ICD-10 codes X60–X84) and comorbidities were defined by their diagnosis within one year before the index date. We also observed whether the patients had a stroke, defined as a primary or secondary diagnosis with the ICD-9 codes 430–437 and ICD-10 codes I60–I67 and G45-G46. Suicide was determined using multiple causes of death with ICD-10 codes X60–X84 and Y870.

### Statistical analysis

Descriptive statistics were adopted to describe the socioeconomic status and other control variables. The chi-square test was performed to compare the education level, sex, age, marital status, CCI, and urbanization level of the region of residence among the groups. A log-rank test^48^ was also used to compare the incidence of stroke, suicide, and death among patients with the catastrophic illness, illness, and control groups. Besides, health conditions, socioeconomic status, and observed medical situations during the year before the index date were also observed. Furthermore, the stratified Cox proportional hazard model was used to explore the risk of stroke, suicide, and death in the three groups after matching after controlling for relevant variables. Hazard ratios (HRs) and 95% confidence intervals (CIs) were calculated. Covariate-adjusted survival curves for stroke, suicide, and death among people with different groups according to the Cox proportional hazard model are presented in the figures. Finally, the between-group differences in the incidence of stroke, suicide, and death per thousand person-years were determined using the univariate Poisson regression test. All statistical analyses were performed using SAS (version 9.4 for Windows; SAS Institute, Cary, NC, USA). A *p*-value of < 0.05 was considered statistically significant.

### Ethics approval and consent to participate

The NHIRD encrypts patient information to protect their privacy and provides researchers with anonymous identification numbers associated with relevant information, including sex, date of birth, medical services received, and prescriptions. Therefore, patient consent is not required to access the NHIRD. The authors assert that all procedures contributing to this work comply with the ethical standards of the relevant national and institutional committees on human experimentation and with the Helsinki Declaration of 1975, as revised in 2008. This retrospective observational study was approved by the Research Ethics Committee of China Medical University Hospital, Taiwan (CMUH109-REC1-016).

### Supplementary Information

Supplementary Figure A.

Supplementary Figure B.

Supplementary Figure C.

Supplementary Legends.

## Data Availability

The data supporting this study’s findings are available from the National Health Insurance Research Database published by the Ministry of Health and Welfare (https://www.mohw.gov.tw/np-108-2.html), Taiwan. Still, restrictions apply to the availability of these data, which were used under license for the current study and are not publicly available. Due to legal regulations imposed by the Taiwanese government related to the Personal Information Protection Act, the database cannot be made publicly available. Furthermore, any raw data are not allowed to be removed from the Health and Welfare Data Science Center. Therefore, the restrictions prohibited the authors from making the minimal data set publicly available. Data are however available from the authors upon reasonable request and with permission of the Health and Welfare Data Science Center, the Ministry of Health and Welfare, Taiwan.
