# Supplementary figures and images for: Stroke and suicide among people with severe mental illnesses
Source: Sci Rep. 2024 Feb 29;14:4991. doi: 10.1038/s41598-024-55564-x (PMC10904760; doi:10.1038/s41598-024-55564-x)

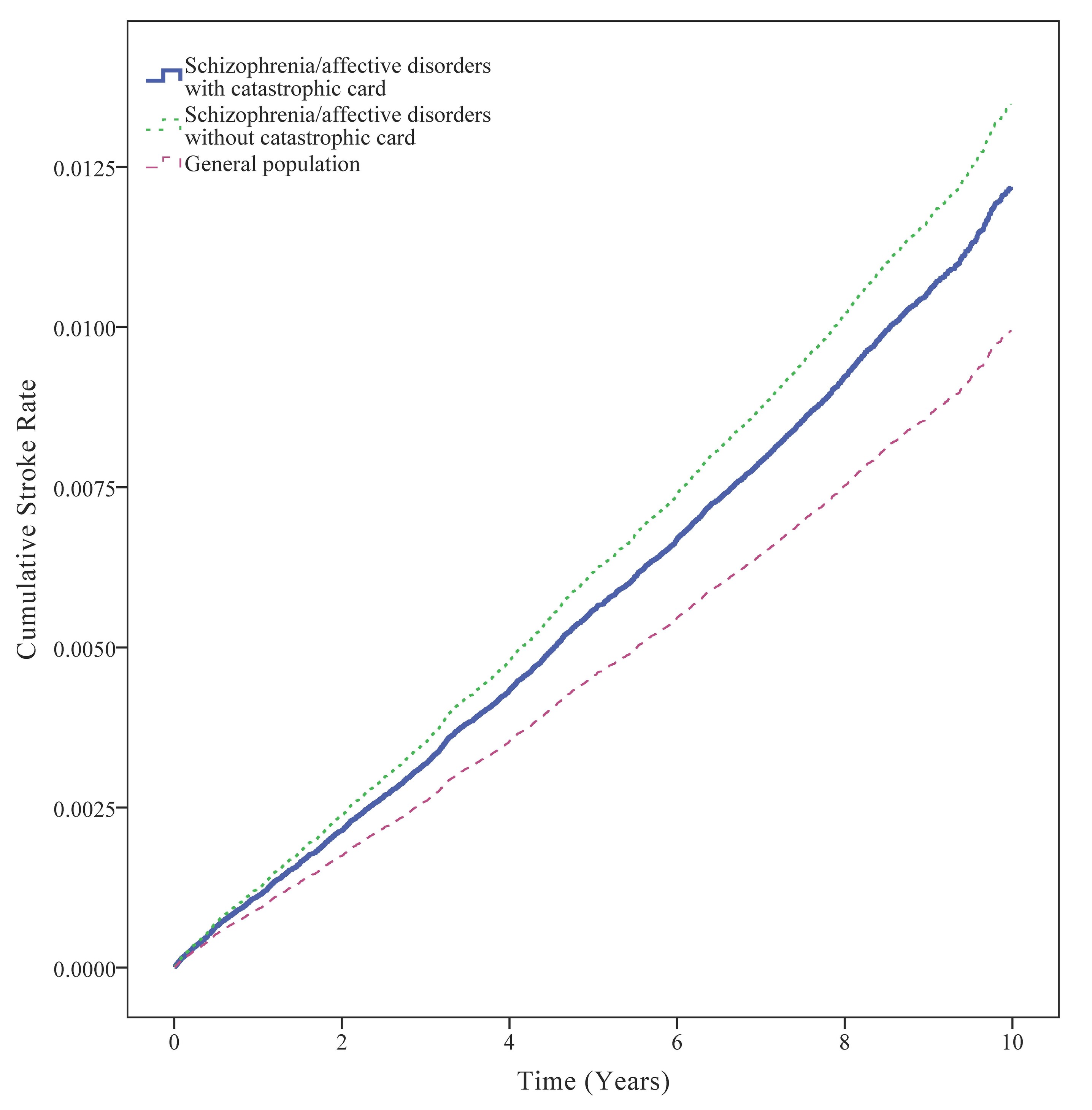

Supplement: Supplementary file 1 — Supplementary Figure A. [file 41598_2024_55564_MOESM1_ESM.jpg]

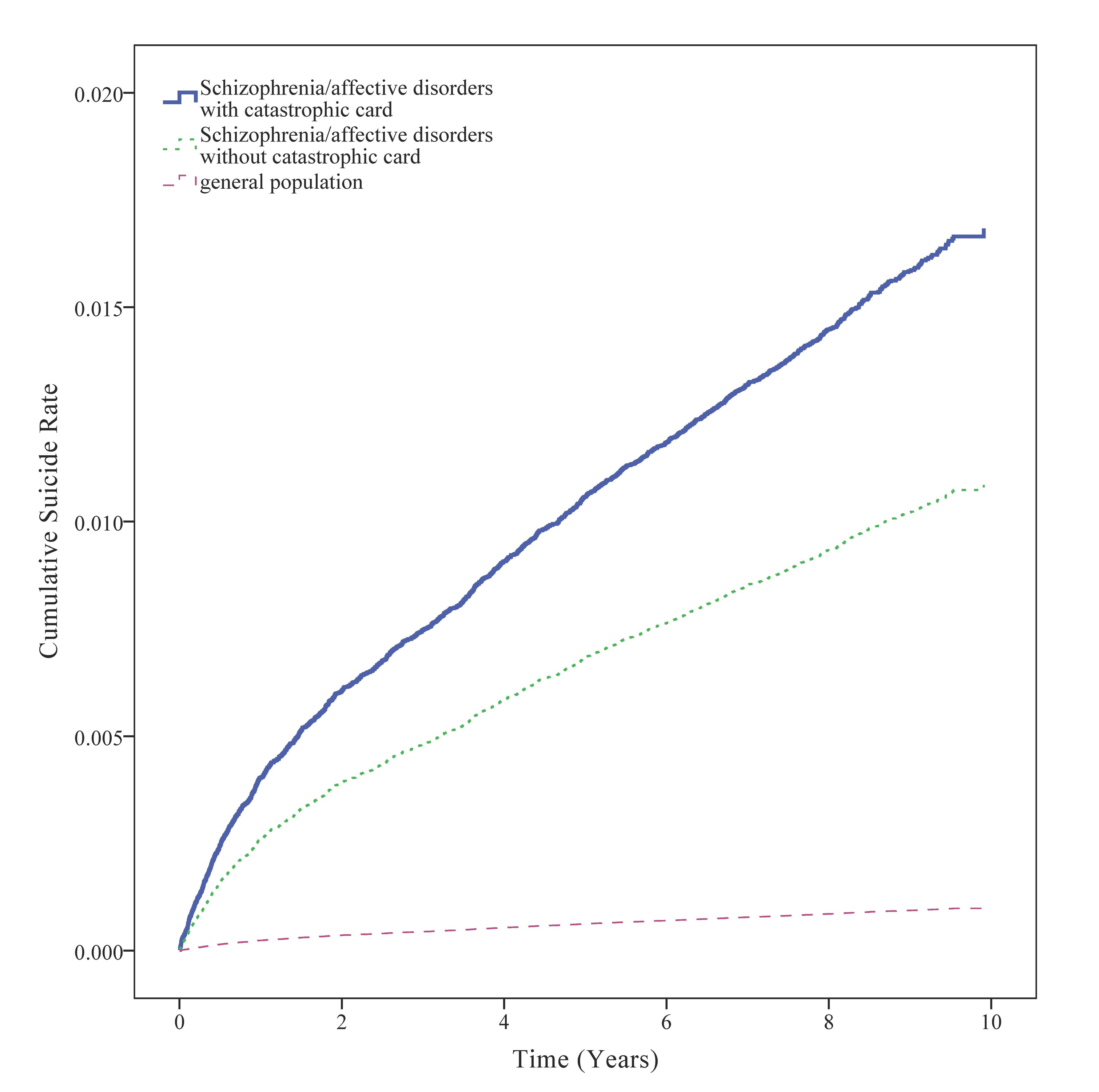

Supplement: Supplementary file 2 — Supplementary Figure B. [file 41598_2024_55564_MOESM2_ESM.jpg]

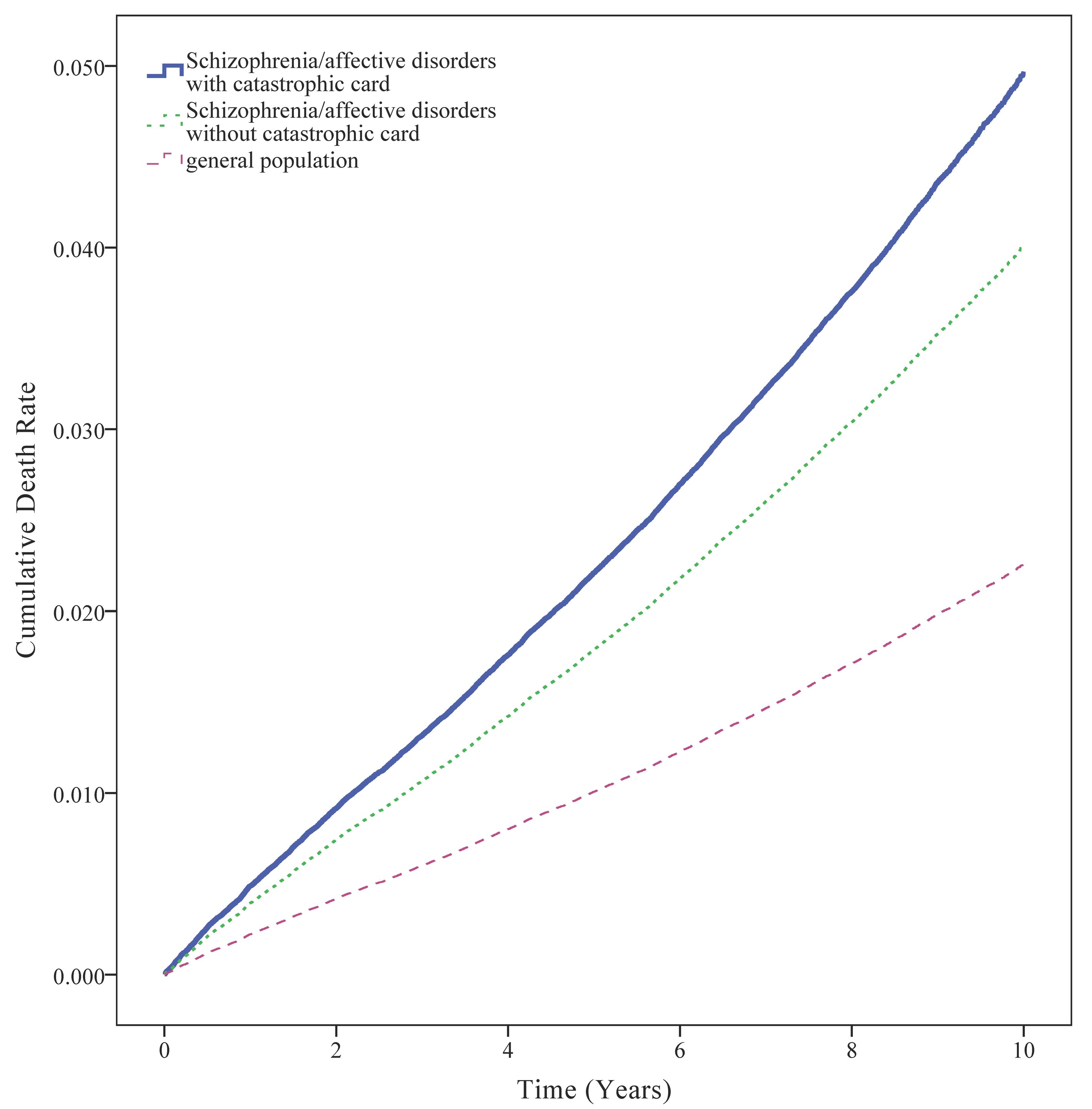

Supplement: Supplementary file 3 — Supplementary Figure C. [file 41598_2024_55564_MOESM3_ESM.jpg]
