# Supplementary material for: Stroke and suicide among people with severe mental illnesses
Source: Sci Rep. 2024 Feb 29;14:4991. doi: 10.1038/s41598-024-55564-x (PMC10904760; doi:10.1038/s41598-024-55564-x)
Supplement: Supplementary file 4 — Supplementary Legends. [file 41598_2024_55564_MOESM4_ESM.docx]

Supplementary Figure A. The covariate-adjusted cumulative incidence curves of stroke among people with SMI compared with the control group.

Supplementary Figure B. The covariate-adjusted cumulative incidence curves of suicide among people with SMI compared with the control group.

Supplementary Figure C. The covariate-adjusted cumulative incidence curves of death among people with SMI compared with the control group.
